# Associations Between Serum Selenium, Zinc, and Copper Levels and Cognitive Function in the Elderly

**DOI:** 10.3390/nu17243872

**Published:** 2025-12-11

**Authors:** Piangporn Charernwat, Sirintorn Chansirikarnjana, Pachara Panpunuan, Piyamitr Sritara, Jintana Sirivarasai

**Affiliations:** 1 Division of Geriatric, Department of Medicine, Faculty of Medicine Ramathibodi Hospital, Mahidol University, Bangkok 10400, Thailand; piangpornc@gmail.com (P.C.); chansirikarn.s@gmail.com (S.C.); 2 Division of Cardiology, Department of Medicine, Faculty of Medicine Ramathibodi Hospital, Mahidol University, Bangkok 10400, Thailand; pachpan@hotmail.com (P.P.); piyamitr.sri@mahidol.ac.th (P.S.); 3 Nutrition Unit, Faculty of Medicine Ramathibodi Hospital, Mahidol University, Bangkok 10400, Thailand

**Keywords:** selenium, zinc, copper, cognitive impairment, Mini-Cog

## Abstract

**Background/Objectives:** Cognitive decline in older people is greatly affected by various risk factors, especially imbalances in trace elements. This study aimed to examine the relationships between serum levels of selenium, zinc, and copper and cognitive impairment. This study included 854 participants aged 63 to 85 years. **Methods:** We conducted clinical assessments of metabolic disorders and measured serum levels of selenium, zinc, and copper. Cognitive impairment was evaluated using the Mini-Cog test. **Results:** The primary analysis identified significant differences (all *p* < 0.05) in age, body mass index, waist circumference, various metabolic parameters (such as fasting plasma glucose, glycated hemoglobin, and plasma triglyceride levels) and some cardiometabolic indices between the groups with and without cognitive impairment. Further assessments using multiple logistic regression and receiver operating characteristic analysis showed an association between increased serum selenium and zinc levels and a protective effect against cognitive impairment. In contrast, elevated serum copper levels were identified as a risk factor for cognitive impairment. This analysis also demonstrated high sensitivity and specificity, along with established cut-off levels for all of the trace elements studied. **Conclusions:** The Mini-Cog test is an effective cognitive screening test for the older population. Our findings establish a significant association between the balanced status of key antioxidant trace elements and cognitive health. Specifically, adequate serum selenium and zinc levels are associated with enhanced cognitive performance, while elevated copper may indicate a pro-oxidant state detrimental to cognitive function. Consequently, these three elements offer promise as practical, accessible biomarkers for the early identification and risk stratification of individuals susceptible to cognitive impairment. Future research should prioritize clinical trials focused on targeted nutritional strategies—specifically optimizing dietary intake and/or supplementation of selenium and zinc while carefully managing copper balance—as a viable primary prevention approach to reduce the global burden of cognitive decline.

## 1. Introduction

The World Health Organization projects that the global population aged 60 years and older will double from 1.06 billion (13.5%) in 2020 to 2.13 billion (22.0%) by 2050 [1]. In Southeast Asia, the older adult population is expected to increase from 77.4 million in 2020 to 173.3 million (22.0%) by 2050 [1]. This rapid demographic shift presents considerable challenges and opportunities for supporting aging communities. The older population faces serious health issues because of chronic diseases and multimorbidity, especially cognitive impairment [2]. The underlying mechanisms of cognitive impairment are complex and multifactorial, involving neurodegeneration, inflammation, and oxidative stress [3]. Recent evidence suggests that trace elements, such as selenium, copper, and zinc, play critical roles in maintaining neuronal health through antioxidant pathways and enzymatic functions [4]. Selenium acts as an important cofactor for antioxidant enzymes, functioning to neutralize toxic free radicals [5]. Zinc is known for its role in synaptic function and neuroprotection, while copper contributes to energy metabolism and neurotransmitter synthesis in the brain [5].

A systematic review that analyzed 44 studies focused on global cognitive functioning showed that higher levels of selenium and zinc were positively correlated with improved cognitive performance among various age groups and demographics [6]. In contrast, elevated copper levels are associated with adverse cognitive outcomes, suggesting that they are a risk factor for cognitive decline [6]. A dietary pattern rich in zinc has been linked to improved cognitive outcomes in Chinese adults, suggesting that zinc plays a protective role against cognitive decline [7]. Additionally, a cross-sectional study involving 416 older adults aged 60 and above from four different regions in China examined the relationship between multi-element combined exposure to selenium (Se) and cognitive function [8]. The findings indicated that higher levels of Se and copper (Cu) were associated with better cognitive function in the elderly. Moreover, selenium may help mitigate cognitive damage caused by calcium [8]. This comprehensive analysis emphasizes the importance of trace elements in cognitive health and highlights the need for further research to examine the mechanisms underlying these relationships. There is growing evidence that dysregulation of trace elements contributes to neurodegeneration, but the specific association between trace element profiles and cognitive impairment is not well understood.

The Mini-Cog test has shown excellent screening characteristics for cognitive decline and dementia in older adults, suggesting that it can be effectively used as a screening tool for the early detection of cognitive decline and dementia, particularly in community settings [9,10,11]. A systematic review and meta-analysis assessed the diagnostic accuracy of the Mini-Cog screening tool for detecting cognitive impairment [12]. This analysis showed that for detecting dementia (based on 6 studies with 4772 participants), the Mini-Cog had a sensitivity of 76% and a specificity of 83%. In the case of mild cognitive impairment (MCI), the Mini-Cog demonstrated a sensitivity of 84% and a specificity of 79%, based on two studies involving 270 participants [12]. In addition, the association between multiple essential trace elements, cognitive impairment with no dementia, and executive function in older Korean adults has been described [13]. Blood manganese (Mn), copper (Cu), zinc (Zn), selenium (Se), and molybdenum (Mo) were measured, and Mn was designated as the most dominant element associated with the cognitive impairment with no dementia, with a U-shaped relationship. Cu and Se levels were positively associated with the percentiles of the Korean color word Stroop test and the frontal/executive tests, including digit symbol coding [13].

Existing literature indicates a relationship between trace elements and cognitive function [9,10,11,12]; however, notable gaps persist that hinder their clinical application and understanding across different regions. The majority of research tends to concentrate on one or two trace elements, resulting in a limited exploration of the independent and potentially confounding interactions among three key elements crucial for the antioxidant defense system: selenium, zinc, and copper. Understanding the balance and interactions among these elements is essential, as an imbalance in one may either be compensated for or may intensify the effects of the others. Furthermore, epidemiological studies examining micronutrients and cognition have predominantly been conducted in Western or North American populations [6], leading to a significant lack of data regarding community-dwelling older adults in Southeast Asia, particularly in Thailand. The unique dietary habits and environmental factors in this region could substantially influence the natural balance and nutritional status of these trace elements. There is an important need for further research that extends beyond only identifying correlations to rigorously assessing the diagnostic utility of these elemental markers. Specifically, it is crucial to evaluate whether a panel of serum elements can serve as practical and accessible biomarkers for early risk stratification in large population screenings.

Therefore, this study aimed to comprehensively investigate the independent and confounding associations between different serum trace element markers, specifically selenium, zinc, and copper, and the risk of cognitive dysfunction classified by the Mini-Cog test in a large community-dwelling population of older individuals in Thailand. Additionally, we aimed to assess the diagnostic utility of these elemental markers as potential biomarkers for identifying individuals at a high risk of cognitive impairment.

## 2. Materials and Methods

### 2.1. Participants

This study involved 854 participants aged 63 to 85 years, with a mean age of 68.45 ± 3.68 years; over 70% of the participants were male, who took part in the Electricity Generating Authority of Thailand cohort study. The initial survey began in 1985 and focused primarily on established risk factors for cardiovascular disease (follow-up surveys were conducted every 5 years). In this study, we used data of the Electricity Generating Authority of Thailand 2012 survey, which provided comprehensive information on the participants’ characteristics and health status. This information was collected through structured questionnaires and health examinations. The assessed variables included age, sex, educational level, occupation, tobacco use, alcohol consumption, physical activity, and a medical and family history.

The cognitive screening process (which was part of the original survey) involved the Mini-Cog assessment, which is a brief and effective tool for evaluating cognitive function. The individuals undergoing this assessment were able to clearly hear and understand the instructions provided by the examiner. Furthermore, the individuals were able to hold a pen and participate in the clock drawing test (CDT), in which they were asked to draw a clock face showing a specific time. This component of the assessment evaluates cognitive and motor skills, providing a comprehensive understanding of an individual’s cognitive function.

The exclusion criteria for this assessment included the following: (1) presence of a major acute medical illness (e.g., active infection, recent stroke within the past 6 months, and unstable cardiac condition); (2) severe cognitive impairment or a pre-existing diagnosis of severe dementia; (3) diagnosis of a major depressive disorder or other psychiatric disorders (e.g., schizophrenia) that severely impair cognition; (4) diseases indicating trace element dyshomeostasis (including a history of Wilson’s disease or Menkes disease) or recent active use of high-dose selenium, zinc, or copper supplements within the past 3 months; and (5) inability to undergo cognitive testing because of severe hearing or vision impairment that prevents administration of the verbal (word recall) and drawing (CDT) components of the Mini-Cog.

The study was conducted with approval from the Ethics Committee of the Faculty of Medicine at Ramathibodi Hospital, affiliated with Mahidol University (COA No. MURA2024/13). Before participation, all individuals involved were thoroughly informed about the study’s purpose, procedures, and potential risks, and written informed consent was obtained from each participant to ensure their understanding and willingness to take part in the research.

### 2.2. Clinical Assessment

A comprehensive clinical assessment involved several important measurements, such as body mass index (BMI); waist circumference (WC), which is an indicator of abdominal fat; systolic blood pressure; and diastolic blood pressure. To enhance the accuracy of our health assessments, systolic blood pressure and diastolic blood pressure were methodically recorded as the average of two separate measurements. These measurements were taken using a calibrated automatic device positioned on the participant’s left arm while they were seated in a comfortable environment. Before the assessment, the participants were encouraged to rest for at least 5 min.

The measurements of height, weight, and waist circumference were conducted by trained personnel following a standardized protocol [14]. Waist circumference was determined at the superior lateral border of the right ilium, recorded to the nearest millimeter. Body Mass Index (BMI) was calculated by dividing weight in kilograms by the square of height in meters. The hip circumference was also measured in centimeters using the same measuring tape at the widest portion of the buttocks, with the tape parallel to the floor. These measurements are important indicators of abdominal fat distribution, which can provide useful insights into an individual’s health risks related to adiposity.

### 2.3. Biochemical Analyses

We collected fasting venous blood samples, these samples were taken at the same time of day for each participant to ensure accurate and reliable results. Using clotted blood, sodium fluoride, and EDTA tubes, we were able to conduct a comprehensive analysis of various biochemical parameters. Following the collection process, the serum samples were efficiently separated and subsequently stored at a temperature of −80 °C, which is crucial for maintaining sample integrity until further analysis. The analysis focused on several important health indicators, including fasting plasma glucose, triglycerides (TGs), total cholesterol, high-density lipoprotein cholesterol (HDL-C), low-density lipoprotein cholesterol, albumin, glycated hemoglobin (HbA1c), blood urea nitrogen, and creatinine. We employed advanced automated methods using the Cobas-Mira system (Roche, Milan, Italy) [15]. This approach was designed to enhance the accuracy and reliability of our results.

Zinc, selenium, and copper levels were measured using inductively coupled plasma mass spectrometry with the Agilent 7700 system (Agilent Technologies, Santa Clara, CA, USA). The protocol for determining serum zinc levels was adapted from a refined method developed by Krachle et al. [16]. To assess serum selenium and copper levels, we used the reference method established by Bumoko et al. [17]. To validate the performance of the inductively coupled plasma mass spectrometry technique, we used Seronorm (Sero AS, Hvalstad, Norway) as a certified reference material, which provided a benchmark for comparison. The accuracy of the measurements, along with the coefficient of variation for the limit of quantification, was fine-tuned to maintain a threshold percentage of <15%. Additionally, the target for the limit of detection was strategically set at half the limit of quantification, which ensured sensitivity in identifying lower concentrations of the analyzed elements.

### 2.4. Metabolic Dysfunction Indices

The metabolic dysfunction indices used in this study represent a comprehensive array of measures specifically designed to evaluate various dimensions of metabolic health, extending beyond the parameters of simple obesity. Among these measures [14], we used the atherogenic index of plasma (AIP), which is a biomarker for cardiovascular risk, reflecting the balance of lipoproteins present in the bloodstream. The lipid accumulation product (LAP), a sex-specific index, integrates WC and triglycerides to provide a continuous estimate of pathologically functional visceral fat. Additionally, the cardiometabolic index (CMI) was calculated using the dyslipidemic ratio with the waist-to-height ratio (WHtR), thereby consolidating measures of fat quality and central distribution into a singular cardiometabolic risk score. Furthermore, the complex sex-specific visceral adiposity index (VAI) was used to assess adipose tissue dysfunction by incorporating WC, BMI, TGs, and HDL-C into a score that is highly predictive of insulin resistance and cardiovascular events. Finally, we used the triglyceride-glucose (TyG) index as a fundamental measure of insulin resistance. This index is derived from the log-transformed product of fasting TG and glucose levels. The applicability of the TyG index was further enhanced by the calculation of TyG-BMI (TyG × BMI) and TyG-WC (TyG × WC) to effectively consider general and central adiposity in the assessment of the overall cardiometabolic burden [18].

The metabolic dysfunction indices were calculated using the following formulas:

AIP = (log TGs/HDL-C)

CMI = (TGs/HDL) × WHtR

LAP for females = (WC − 58) × TGs (mmol/L)

LAP for males = (WC − 65) × TGs (mmol/L)

VAI for females = WC/(36.58 + [1.89 × BMI]) × TGs/0.81 × 1.52/HDL-C

VAI for males = WC/(39.68 + [1.88 × BMI]) × TGs/1.03 × 1.31/HDL-C

TyG = Ln(TGs [mg/dL] × fasting glucose [mg/dL]/2)

TyG-BMI = TyG × BMI

TyG-WC = TyG × WC

### 2.5. Mini-Cog Assessment

The Mini-Cog assessment is a highly useful and effective method for the rapid, initial screening of cognitive function, particularly in primary care and community settings [9,10]. It is designed to be quick (taking approximately 3 min) and easy to administer, making it a practical tool for identifying potential cognitive impairment that warrants further diagnostic evaluation.

#### 2.5.1. Three-Item Recall Test (Memory Component)

In this part, the participants were introduced to three unrelated words that they needed to memorize. After a brief interval, they were encouraged to recall these words without any prompts, allowing for a straightforward evaluation of their memory retention through uncued three-word recall. The participants received a score ranging from 0 to 3, in accordance with the accuracy of their recalled words. A score of 3 indicated complete recall with strong memory function, while a score of 0 suggested potential areas for improvement in memory retention.

#### 2.5.2. CDT Component

In this interactive component, the participants drew an analog clock face, including all 12-h markers. They were also asked to set the clock hands at a specified time, such as 11:10 [19]. This task assessed not only their ability to visualize time but also their spatial awareness and organizational skills. The CDT was evaluated using a clear binary system. A drawing was considered normal if it presented all numbers in their correct positions and had hands showing the correct time, indicating good cognitive functioning [19]. If the drawing did not meet these criteria, it was classified as abnormal, highlighting potential areas of concern that may warrant further exploration.

In addition to these individual components, in the Mini-Cog assessment, we applied a structured scoring model, as proposed by Borson et al. [9,10], to interpret the results meaningfully. This integrated approach allowed for a comprehensive assessment of dementia or cognitive impairment, starting with the evaluation of a three-item recall score [9,10]. A score of 3 suggested that the individual likely had intact memory function and did not show signs of dementia. A score of 0 indicated that the individual had dementia, suggesting a need for support in memory. A score of 1 or 2 represented an inconclusive result, prompting further evaluation through the CDT, ensuring a thorough approach to assessment. If the CDT result was “normal,” the individual was classified as having no signs of dementia, indicating preserved cognitive abilities. If the CDT result was “abnormal,” the individual was considered to have dementia [9,10], and the results guide necessary interventions and support.

By using this algorithmic approach, the participants were effectively categorized into two groups: The Cognitive Impairment Group (CI Group): Composed of participants who scored 0 to 2 on the Mini-Cog assessment [9,10], indicating a positive screen for potential cognitive impairment. The Non-Cognitive Impairment Group (Non-CI Group): Composed of participants who scored 3 to 5 on the Mini-Cog assessment, indicating a negative screen for significant cognitive impairment [9,10].

### 2.6. Statistical Analysis

Data analysis was conducted using the Statistical Package for Social Sciences version 25 (IBM Corp., Armonk, NY, USA). To assess the normality of continuous variables, histograms and the Kolmogorov–Smirnov test were used. Serum trace elements were log-transformed and presented as geometric means. Continuous variables are presented as the mean with standard deviation, while categorical variables are shown as the frequency with percentage. Comparisons of sociodemographic variables, lifestyle habits, and anthropometric and biochemical measurements between participants in the cognitive impairment and non-cognitive impairment groups were performed using Pearson’s chi-square test for categorical variables and the independent samples Student’s *t*-test for continuous variables. Statistical significance was defined as a *p*-value less than 0.05.

We assessed the risk factors for cognitive impairment through a series of analyses using univariate and multivariable logistic regression. First, univariate logistic regression was used to identify the unadjusted relationships between each independent variable, such as serum trace elements and metabolic dysfunction indices, and cognitive impairment, as indicated by the Mini-Cog score. We then performed multivariable logistic regression to determine the independent contributions of key factors and focused on trace elements (zinc, selenium, and copper), while accounting for confounding variables. The final model included age, education level, smoking status (current, former, or never), and a family history of hypertension or diabetes mellitus. The results are presented as odds ratios (ORs) and adjusted ORs, along with 95% confidence intervals (CIs) and *p* values to quantify the risk.

The receiver operating characteristic (ROC) curve was constructed to assess the performance of serum trace elements in predicting cognitive impairment. This analysis allowed for the calculation of the area under the curve values, accompanied by 95% CIs, which provided a measure of the model’s accuracy. Additionally, sensitivity and specificity were determined for each obesity index, offering insights into its diagnostic effectiveness. To establish cut-off points for these serum trace elements, the Youden index was used. This index, which was calculated as the sum of sensitivity and specificity minus one, helps identify the optimal balance between true positive and true negative rates. The cut-off value associated with the highest Youden index was selected as the most effective threshold for diagnosing cognitive impairment.

## 3. Results

Demographic and lifestyle characteristics in the non-cognitive impairment (*n* = 563) and cognitive impairment (*n* = 291) groups are shown in Table 1. The mean age of the participants was significantly higher in the cognitive impairment group (69.16 ± 4.69 years) than in the non-cognitive impairment group (67.40 ± 4.41 years). Most participants in both groups were men. There was a difference in educational level between the two groups. A higher proportion of individuals in the cognitive impairment group had <12 years of education compared to those in the non-cognitive impairment group (32.3% vs. 23.6%, *p* < 0.05). No significant differences in smoking status, alcohol consumption, or physical activity were observed between the two groups.

Regarding anthropometric measurements, BMI and WC were significantly higher in the cognitive impairment group than in the non-cognitive impairment group (*p* < 0.05). However, there were no differences in blood pressure between the two groups. The prevalence of most medical risk factors was similar between the groups. A family history of dementia was slightly more common (*p* > 0.05) in the cognitive impairment group than in the non-cognitive impairment group (9.3% vs. 7.8%). Additionally, >40% of individuals in both groups were using antihypertensive and lipid-lowering medications. Finally, in the three-item recall test, the cognitive impairment group showed a significantly higher rate of abnormal scores than the non-cognitive impairment group (16.8% vs. 0.4%).

Table 2 shows details of biochemical parameters related to blood glucose levels, lipid profiles, and kidney function. Fasting plasma glucose and HbA1c levels were significantly higher in the cognitive impairment group than in the non-cognitive impairment group. Regarding the lipid profile, triglyceride levels were higher in the cognitive impairment group than in the non-cognitive impairment group (147.71 ± 85.21 vs. 104.79 ± 39.43 mg/dL) (*p* < 0.05). There was no significant difference in blood urea nitrogen or creatinine levels between the two groups.

A comprehensive analysis of cardiometabolic indices among individuals categorized as low and high risk for cognitive impairment showed differences in the markers associated with lipid metabolism and insulin resistance. Specifically, key indicators, such as the AIP, CMI, LAP, and TyG index, were significantly higher in the cognitive impairment group than in the non-cognitive impairment group. In contrast, TyG-BMI, TyG-WC, and the VAI were not significantly different between the two groups.

The comparative analysis of serum trace element levels showed a distinct pattern related to the risk of cognitive impairment. The cognitive impairment group showed significantly lower mean levels of serum selenium (93.81 ± 25.20 vs. 139.02 ± 39.49 µg/L) and serum zinc (515.59 ± 195.94 vs. 890.15 ± 221.34 µg/L) than the non-cognitive impairment group. In contrast, the cognitive impairment group showed a notably higher mean serum copper level than the non-cognitive impairment group (1224.99 ± 244.23 vs. 666.88 ± 163.81 µg/L, *p* < 0.05).

The unadjusted OR analysis of cognitive impairment, age, and educational level showed significant associations (Table 3). Specifically, the odds of having cognitive impairment increased by 4% for every 1-year increase in age (OR = 1.04, 95% CI: 1.01–1.07, *p* = 0.01). Individuals with less than 12 years of education had $1.55$ times the odds of screening positive for cognitive impairment (i.e., being in the Mini-Cog Cognitive Impairment Group) compared with those who had 12 or more years of education (OR = 1.55, 95% CI: 1.13–2.21, *p* = 0.001). Other factors were not found to have a significant unadjusted association with cognitive impairment. The unadjusted analysis showed that several obesity markers, including BMI and WC, along with indicators of glycemic control, such as fasting plasma glucose and HbA1c, as well as triglyceride levels, were significantly associated with a higher risk of developing cognitive impairment. Comprehensive indices, including the AIP and CMI, showed a positive association with cognitive impairment (*p* < 0.05). Additionally, serum copper (OR = 1.44, 95% CI: 1.24–1.67, *p* = 0.001) and selenium (OR = 0.64, 95% CI: 0.57–0.83, *p* = 0.001) levels were positively associated with cognitive impairment, and serum zinc levels were negatively associated with cognitive impairment (OR = 0.72, 95% CI: 0.57–0.90, *p* = 0.001).

In the multivariate logistic regression analysis, we identified key independent risk factors for cognitive impairment (Table 4). The adjusted model showed that serum copper levels were associated with a slightly increased risk of cognitive impairment (*p* < 0.05). However, serum selenium and zinc levels were highly significant protective factors against cognitive impairment. All other factors, including traditional cardiometabolic risk markers, were not independently associated with cognitive impairment after controlling for sex, smoking habits, alcohol consumption, physical activity, medical history, and medication use.

**Figure 1:**
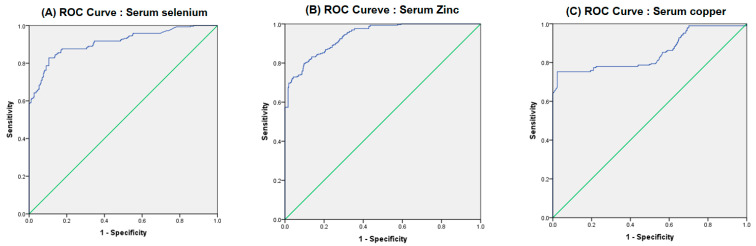
ROC curves for identifying cognitive impairment according to the serum trace elements. (For each figure, the green line is the reference line and the blue line represents its diagnostic performance).

Following the identification of independent risk factors by the adjusted OR analysis, we performed the ROC curve analysis (Figure 1 and Table 5). The ROC curve provides valuable insights into the relationship between Sensitivity (True Positive Rate) and 1-Specificity (False Positive Rate) for various cut-off values of serum selenium, zinc, and copper, as illustrated in Figure 1A, Figure 1B, and Figure 1C, respectively. The blue line indicates the diagnostic performance of each trace element, while the green diagonal line marks the threshold for no discrimination, corresponding to an Area Under the Curve (AUC) of 0.5, which suggests that a test is no more effective than random chance. In this study, the observed ROC curves are predominantly located in the upper-left corner, suggesting that these serum trace elements can be effective classifiers for cognitive impairment. The calculated AUC values reflect this potential, with 0.911 for serum selenium, 0.938 for serum zinc, and 0.853 for serum copper, all of which demonstrate strong discriminatory power. This evidence supports further exploration and application of these serum trace elements in clinical assessments of cognitive health.

Table 5 shows the quantitative results of the ROC curve analysis, which indicate the diagnostic accuracy of the three trace elements as predictors of cognitive impairment. Zinc had the highest area under the curve (0.938) and was thus the best overall marker for distinguishing between individuals with cognitive impairment and those without cognitive impairment. The optimal cut-off of 693.43 µg/L for zinc correctly identified 80% of true cases and ruled out 89% of non-cases. Selenium provided the highest sensitivity (82%), with an optimal cut-off of 114.56 µg/L, which indicated that it was the most effective element for correctly identifying individuals who actually have cognitive impairment. Copper demonstrated an impressive specificity of 96%, which indicated that when a patient’s copper level is below the high-risk threshold of 898.110 µg/L, we can be confident that there is no cognitive impairment. However, the sensitivity of copper was low at 75%, which suggested that some cases of cognitive impairment may be missed.

## 4. Discussion

The Mini-Cog is an effective screening tool for the early detection of cognitive decline and dementia in older adults [20]. Previous study indicated that this cognitive test demonstrated high utility for detection, with a low false-negative rate for MCI and a low false-positive rate for dementia. [11]. In this study, 34.1% of the participants showed cognitive impairment using the Mini-Cog tool. This finding is similar to that of a study in India, which reported a 44.2% prevalence among older individuals with an average age of 66.02 years [21]. Another study showed that the prevalence of dementia based on the Mini-Cog ranged from 32.2% to 87.3% [22].

Age and educational level play important roles in cognitive decline, as supported by our results and previous studies [23,24,25,26]. Aging is the main risk factor for cognitive decline, increasing the likelihood of MCI and dementia [23]. The Longitudinal Aging Study Amsterdam, which involved 2527 cognitively healthy participants aged 55–85 years, showed a significant nonlinear relationship (*p* < 0.05), indicating that cognitive decline becomes more pronounced with age, especially after 70 years of age [24]. The aging process in the brain drives cognitive decline through several interrelated changes, including brain atrophy (particularly volume loss in memory and executive function centers), alterations in key neurotransmitters (such as acetylcholine and monoamines), cellular and molecular aging (marked by mitochondrial dysfunction, accumulation of oxidatively damaged molecules, and altered gene expression), and dysregulation of neuronal calcium homeostasis [25]. An association between a low level of education and cognitive impairment has been proposed, which is primarily explained by the theory of cognitive reserve. Level of education is widely used as an indicator of cognitive reserve. Individuals with a higher reserve are able to tolerate a higher neuropathological burden than those with a lower reserve [26].

This study provides important insights into the nutritional status of two distinct populations: individuals with cognitive impairment and those without. We conducted a comprehensive analysis of anthropometric data and assessed the prevalence of hypertension, type 2 diabetes mellitus (T2DM), and dyslipidemia—key indicators of potential metabolic diseases associated with nutrition. Our findings indicate that individuals with cognitive impairment display significantly higher body mass index (BMI) and waist circumference (WC) compared to their non-cognitive impairment counterparts (*p* < 0.05). This suggests a greater burden of obesity and central adiposity in the cognitively impaired population, warranting further investigation and targeted interventions. Conversely, we observed no significant differences in the prevalence of T2DM and dyslipidemia between the two groups. This observation suggests further exploration into the complex relationship between cognitive impairment and metabolic health, emphasizing the need for additional research in this area.

Our findings suggest that important metabolic risk factors, such as obesity, elevated plasma glucose levels, high HbA1c values, and increased triglyceride levels, are associated with cognitive impairment. Notably, a high BMI and an increased WC (indication of abdominal or central obesity) can greatly contribute to the risk of cognitive impairment [27,28,29]. The proposed mechanisms related to these risk factors involve neuroinflammation. Adipocyte hypertrophy and hyperplasia, as well as an increase in free fatty acid levels, lead to the process of immune cell recruitment (e.g., macrophages) and the release of a cascade of adipokines and pro-inflammatory cytokines [27]. Furthermore, the main cytokines, including interleukin-1β, tumor necrosis factor-α, and interleukin-6, and the activation of microglia and astrocytes lead to the disruption of the blood–brain barrier and to cognitive impairment [28]. A study involving 17,000 individuals aged 65–98 years showed significant associations (*p* < 0.05) between abdominal adiposity, lean body mass, and fat body mass and dementia and cognitive decline. Specifically, a positive relationship was observed between the WC-to-BMI ratio and the risk of dementia and cognitive deterioration [29].

Hypertriglyceridemia is a risk factor for cognitive impairment and dementia, particularly vascular dementia [30]. A study of 125,727 individuals aged 46–67 years showed that moderate hypertriglyceridemia was associated with a higher risk of non-Alzheimer’s dementia and ischemic stroke. This association, which was adjusted for age, sex, and apolipoprotein E genotype, indicated the role of plasma triglycerides as a common risk factor for dementia and atherosclerotic cardiovascular disease [30]. The principal mechanism for this association may involve cerebrovascular dysfunction and small vessel disease (through atherosclerosis and arterial stiffness, endothelial dysfunction, and blood–brain barrier damage), neuroinflammation, oxidative stress–induced synaptic loss, and neuronal cell death [31].

Cognitive impairment is a neurodegenerative condition involving metabolic factors. This study evaluated cardiometabolic indices related to insulin resistance and atherogenic dyslipidemia, which are risk factors for cardiovascular disease–related dementia. We found that the AIP, CMI, LAP, and TyG indices were higher in participants with cognitive impairment than in those without cognitive impairment. The univariate analysis showed that the AIP and CMI were significant predictors of cognitive impairment. However, after adjusting for factors such as sex, smoking, and physical activity, these associations lost statistical significance. A study of 3170 participants (average age: 56.5 years, 53.8% men) showed that a high AIP was associated with cerebral small vessel disease [32]. Common imaging signs of cerebral small vessel disease include white matter hyperintensities and lacunar infarcts, which are leading causes of vascular cognitive impairment [32]. The CMI is associated with higher risks of hypertension, diabetes, metabolic syndrome, and stroke, largely due to central obesity and atherogenic dyslipidemia [33]. An elevated CMI can cause systemic inflammation and vascular damage, accelerating neurodegeneration and cognitive decline [34]. The LAP is strongly associated with insulin sensitivity in patients with impaired glucose tolerance or type 2 diabetes and effectively identifies subclinical vascular damage [35]. The TyG index correlates well with insulin resistance and diabetes onset [36]. In a 16-year study of 8511 participants (average age: 51.9 years), the TyG index was more predictive of cardiovascular diseases than the homeostasis model assessment of insulin resistance [37]. Disrupted insulin signaling makes neurons vulnerable to metabolic stress, leading to neuronal dysfunction, decreased cognitive ability, and an increased risk of dementia [38].

Trace elements play a major role in the development of various neurodegenerative diseases [39]. These elements are crucial for normal brain function as essential cofactors for a variety of enzymatic reactions, thereby directly affecting the regulation of oxidative stress [40], synthesis of neurotransmitters [41], and proper folding of proteins [42], but they can contribute to pathological processes when their homeostasis is disrupted. The high sensitivity and specificity observed in the ROC analysis for the studied trace elements confirmed their vital role in cognitive health. Specifically, the data highlight zinc, copper, and selenium as strong indicators of cognitive status. Notably, our study suggested that selenium and zinc provide protective benefits, while copper appears to contribute to an increased risk of cognitive impairment, similar to the findings of previous studies [43,44,45]. Higher plasma zinc levels and a higher zinc/copper ratio were associated with lower odds of MCI in patients with type 2 diabetes, while higher copper levels increased the risk of MCI [43]. A study using the National Health and Nutrition Examination Survey (NHANES) data of 3042 adults aged ≥60 years assessed cognitive performance using tests of immediate, delayed, and working memory [44]. This previous study showed that higher selenium levels were strongly associated with better performance on all tests, even after adjusting for factors such as age, sex, and health conditions. A longitudinal study examined very old adults to examine the association between selenium status and cognitive decline over 3–5 years [45]. Initially, optimal serum selenium levels were positively associated with overall cognitive function, as measured by the Mini-Mental State Examination score, but this association did not persist over time.

Zinc is essential in neurodegenerative diseases and acts as a cofactor for enzymes involved in the antioxidant defense system. Zinc protects cells from oxidative damage, stabilizes cell membranes, inhibits the enzyme NADPH oxidase, and stimulates the production of metallothioneins, which effectively reduce hydroxyl radicals and sequester reactive oxygen species in metabolic disease–induced oxidative stress [46]. Zinc plays a crucial role in various brain functions, including affecting neurotransmission and sensory processing and activating pro-survival and pro-death neuronal signaling pathways [47]. The role of selenium and selenoproteins regarding cognitive function may involve many mechanisms, such as the dopamine pathway, the acetylcholine pathway, oxidative stress, neuroinflammation, calcium homeostasis, and brain cholesterol metabolism [48]. Copper is involved in numerous physiological processes, such as antioxidant enzyme activity, neuropeptide activation, the synthesis of connective tissues, and neurotransmission. Additionally, copper regulates intracellular signal transduction, balances catecholamines, facilitates the myelination of neurons, and ensures efficient synaptic transmission within the central nervous system [49]. However, high copper levels can lead to cellular dyshomeostasis through redox-active transition metal–induced oxidative stress, mitochondrial dysfunction, apoptosis, and autophagy [45].

In the context of this population-based study, the finding of compromised antioxidant status (imbalances in selenium, zinc, and copper) in the cognitive impairment group suggests a potential role of primary defense against ROS and RNS. This persistent oxidative environment, frequently associated with metabolic conditions such as T2DM and obesity, is a well-established driver of molecular injury. While we did not directly measure the resulting cellular damage, this compromised defense system is known to accelerate damage to major cellular components, including lipids, proteins, and DNA (DNA damage) [40]. We infer that the observed trace element levels in our study population can lead to the neurodegenerative process by heightening the individual’s susceptibility to cumulative, long-term oxidative damage.

While the role of trace element dyshomeostasis in neurodegeneration is widely accepted, the diagnostic performance and clinical utility of circulating levels of copper, zinc, and selenium for identifying older patients with cognitive impairment have yet to be systematically quantified. A key strength of this study lies in its novel application of diagnostic accuracy analysis (via ROC curves) to assess the clinical utility of these nutritional biomarkers. To the best of our knowledge, this work is the first to systematically quantify the predictive value of plasma copper, zinc, and selenium incorporated into routine geriatric screening. These findings, demonstrating high sensitivity and specificity, are essential for creating accessible, nutrition-based screening strategies to assess the risk of cognitive decline, thereby emphasizing the clinical translational potential of these markers. However, our study has several methodological limitations that necessitate further research. Primarily, the cross-sectional design establishes an association between trace element status and cognitive impairment but cannot infer causation. Therefore, longitudinal studies are required to confirm this relationship. Furthermore, a significant limitation is the lack of measurements for trace element-related binding proteins such as ceruloplasmin (for copper), selenoproteins (for selenium), and metallothioneins (for zinc). The inclusion of these proteins is crucial in future research, as measuring only the free circulating element is less mechanistically informative. Quantifying these bound forms would significantly enhance the predictive power and clarify the mechanistic relevance of these nutritional markers in cognitive impairment. An additional limitation of this study is the sex distribution of the cohort, which consisted of a higher proportion of male participants (70% male compared to 30% female). This demographic imbalance is significant because there are known sex-based differences in the metabolism of trace elements such as iron, copper, and selenium [50,51], as well as in the prevalence, manifestation, and progression of cognitive impairment [52,53]. As a result, the associations observed between selenium, zinc, copper, and cognitive performance may not be fully applicable to a population with a greater proportion of female participants, thus limiting the exploration of these potential differences.

## 5. Conclusions

This study shows associations between a range of factors, including demographic, clinical, and biochemical parameters, and the levels of serum selenium, zinc, and copper in relation to cognitive impairment in the older Thai population. Notably, in the final model using logistic regression and ROC analysis, only the levels of these three trace elements were significant biomarkers for identifying the risk of cognitive impairment. These results strongly suggest that nutritional biomarkers should be incorporated into routine clinical practice. This approach could facilitate early identification and targeted therapeutic interventions, such as zinc supplementation, aimed at restoring essential metal homeostasis in the aging population.

## Figures and Tables

**Table 1:** General parameters of study population (*n* = 854).

	Non-Cognitive Impairment (*n* = 563)	Cognitive Impairment (*n* = 291)
Age, years	67.40 ± 4.41	69.16 ± 4.69 *
Sex, *n* (%)		
Male	416 (73.9)	225 (77.3)
Female	147 (26.1)	66 (22.7)
Educational level, *n* (%)		
<12 years	133 (23.6)	94 (32.3) *
≥12 years	430 (76.4)	197 (67.7)
Smoking status, *n* (%)		
Non-smoker	319 (56.7)	157 (54.0)
Former-smoker	200 (35.5)	114 (39.2)
Current smoker	44 (7.8)	20 (6.9)
Alcohol consumption, *n* (%)		
Yes	193 (34.3)	98 (33.7)
No	370 (65.7)	193 (66.3)
Physical activity, *n* (%)		
Yes		
No		
SBP, mmHg	133.69 ± 18.76	132.83 ± 17.41
DBP, mmHg	77.71 ± 10.06	76.52 ± 10.35
BMI, kg/m^2^	23.33 ± 3.01	26.97 ± 3.46 *
WC, cm	84.79 ± 8.92	95.20 ± 7.89 *
Medical risk factor, *n* (%)		
Hypertension	301 (53.5)	172 (59.1)
Type 2 diabetes	106 (18.9)	60 (20.6)
Dyslipidemia	366 (64.1)	179 (61.5)
History of Stroke/Transient Ischemic Attack (TIA)	-	-
Sleep disorder	256 (45.5)	132 (45.4)
Family history of dementia	44 (7.8)	27 (9.3)
Medication use, *n* (%)		
Antihypertensive	265 (47.1)	148 (50.9)
Antidiabetic	92 (16.3)	50 (17.2)
Lowering-lipid	244 (43.3)	115 (39.5)
Antiplatelet/Anticoagulant	96 (17.1)	42 (14.4)
Cognitive assessment, *n* (%)		
CDT-Normal	462 (92.1)	270 (92.8) *
CDT-Abnormal	101 (17.9)	21 (7.0)
3-Item recall-Normal	561 (99.6)	242 (83.2)
3-Item recall-Abnormal	2 (0.4)	49 (16.8)

Data are presented as mean ± SD, or number (percent) as indicated. * Significantly different from non-cognitive impairment group with *p* values < 0.05. BMI: body mass index; DBP: diastolic blood pressure; SBP: systolic blood pressure; WC: waist circumference.

**Table 2:** Biochemical parameters and serum trace element levels among both study groups.

	Non-Cognitive Impairment (*n* = 563)	Cognitive Impairment (*n =* 291)
FPG, mg/dL	93.26 ± 12.47	107.21 ± 26.91 *
HbA1C, %	5.78 ± 0.43	6.17 ± 0.92 *
TC, mg/dL	206.31 ± 43.23	202.63 ± 42.61
TG, mg/dL	104.79 ± 39.43	147.71 ± 85.21 *
HDL-C, mg/dL	59.74 ± 16.63	58.51 ± 15.07
LDL-C, mg/dL	134.11 ± 40.06	131.63 ± 38.31
Albumin, mg/dL	4.55 ± 0.24	4.55 ± 0.23
BUN, mg/dL	14.89 ± 3.99	14.72 ± 4.12
Creatinine, mg/dL	0.99 ± 0.35	0.98 ± 0.23
Cardiometabolic indices		
AIP	0.42 ± 0.31	0.64 ± 0.48 *
CMI, mmol/L	0.55 ± 0.32	0.77 ± 0.46 *
LAP, mmol/L	31.89 ± 12.08	41.17 ± 24.26 *
TyG	6.97 ± 0.32	7.16 ± 0.34 *
TyG-BMI	173.87 ± 29.55	176.43 ± 23.33
TyG-WC	624.53 ± 86.54	632.15 ± 39.76
VAI, mmol/L	1.99 ± 0.74	2.02 ± 0.42
Serum selenium, µg/L ^a^	139.02 ± 39.49	93.81 ± 25.20 *
Serum zinc, µg/L ^a^	890.15 ± 221.34	515.59 ± 195.94 *
Serum copper, µg/L ^a^	666.88 ± 163.81	1224.99 ± 244.23 *

Data are presented as mean ± SD, or number (percent) as indicated. ^a^ geometric mean; * Significantly different from non-cognitive impairment group with *p* value < 0.05. AIP: atherogenic index of plasma; BUN: Blood urea nitrogen; CMI: cardiometabolic index; FPG: Fasting plasma glucose; HbA1C: glycated hemoglobin; HDL-C: high-density lipoprotein cholesterol; LAP: lipid accumulation product; LDL-C: low-density lipoprotein cholesterol; TC: total plasma cholesterol; TG: triglycerides; TG/HDL-C; triglyceride to HDL-C ratio; TyG: triglyceride/glucose index; TyG-BMI: triglyceride-adjusted body mass index; TyG-WC: triglyceride-adjusted waist circumference index; VAI: visceral adiposity index.

**Table 3:** Univariate analysis of serum trace elements and associated factors with cognitive impairment by Mini-Cog.

	Unadjusted Odds Ratio (OR)	95% Confidence Interval (CI)	*p*-Value
BMI, kg/m^2^	1.28	1.18–1.39	0.00
WC, cm	1.19	1.08–1.15	0.00
FPG, mg/dL	1.03	1.02–1.05	0.00
HbA1C, %	1.85	1.22–2.81	0.00
TC, mg/dL	0.97	0.94–1.01	0.07
TG, mg/dL	1.02	1.01–1.02	0.00
HDL-C, mg/dL	1.04	0.99–1.07	0.08
LDL-C, mg/dL	1.03	0.99–1.06	0.07
Albumin, mg/dL	1.11	0.43–2.81	0.83
Cardiometabolic indices			
AIP	1.54	1.04–2.48	0.03
CMI, mmol/L	1.39	1.06–1.81	0.01
LAP, mmol/L	1.25	0.35–3.68	0.73
TyG	1.11	0.49–2.75	0.83
TyG-BMI	1.45	0.74–3.08	0.39
TyG-WC	1.30	0.90–1.87	0.17
VAI, mmol/L	1.03	0.70–1.51	0.90
Serum selenium, µg/L ^a^	0.64	0.57–0.83	0.00
Serum zinc, µg/L ^a^	0.72	0.57–0.90	0.00
Serum copper, µg/L ^a^	1.44	1.24–1.67	0.00

Data are presented as mean ± SD, or number (percent) as indicated. ^a^ geometric mean; AIP: atherogenic index of plasma; BMI: Body mass index; CMI: cardiometabolic index; FPG: Fasting plasma glucose; HbA1C: glycated hemoglobin; HDL-C: high-density lipoprotein cholesterol; LAP: lipid accumulation product; LDL-C: low-density lipoprotein cholesterol; TC: total plasma cholesterol; TG: triglycerides; TG/HDL-C: triglyceride to HDL-C ratio; TyG: triglyceride/glucose index; TyG-BMI: triglyceride-adjusted body mass index; TyG-WC: triglyceride-adjusted waist circumference index; VAI: visceral adiposity index; WC: Waist cimcumference.

**Table 4:** Multivariate analysis of serum trace elements and associated factors with cognitive impairment by Mini-Cog.

	Adjusted Odds Ratio * (OR)	95% Confidence Interval (CI)	*p*-Value
Age, years	1.08	0.94–2.14	0.09
Educational level: <12 years	1.05	0.66–7.72	0.87
BMI, kg/m^2^	1.41	0.85–2.35	0.17
WC, cm	1.09	0.91–1.32	0.34
FPG, mg/dL	1.06	0.98–1.15	0.13
HbA1C, %	1.51	0.13–6.96	0.76
TG, mg/dL	1.02	0.99–1.05	0.06
Cardiometabolic indices			
AIP	3.09	0.30–7.62	0.34
CMI, mmol/L	1.21	0.59–9.89	0.79
Serum selenium, µg/L ^a^	0.82	0.74–0.91	0.00
Serum zinc, µg/L ^a^	0.95	0.93–0.98	0.00
Serum copper, µg/L ^a^	1.01	1.00–1.02	0.00

Data are presented as mean ± SD, or number (percent) as indicated. ^a^ geometric mean; * Significantly different from non-cognitive impairment group with *p* value < 0.05. AIP: atherogenic index of plasma; BMI: Body mass index; CMI: cardiometabolic index; FPG: Fasting plasma glucose; HbA1C: glycated hemoglobin; LAP: lipid accumulation product; TG: triglycerides; WC: Waist cimcumference.

**Table 5:** The optimal cut-off values and diagnostic performance metrics of serum trace elements associated with cognitive impairment.

Trace Element Predictor	AUC (95% CI)	*p*-Value	Optimal Cut-Off Value (Units)	Sensitivity (%)	Specificity (%)
Selenium	0.911 (0.888–0.933)	0.000	≤114.56 µg/L	82	88
Zinc	0.938 (0.922–0.953)	0.008	≤693.43 µg/L	80	89
Copper	0.853 (0.822–0.885)	0.000	≥898.110 µg/L	75	96

## Data Availability

The data used to support the findings of this study can be made available by the corresponding author upon request due to ethical restrictions.
